# Academic women’s negotiation of gender identities in non-elite Chinese universities

**DOI:** 10.3389/fpsyg.2023.1083203

**Published:** 2023-03-22

**Authors:** Boya Yuan, Xiaoming Tian

**Affiliations:** ^1^Faculty of Education and Social Work, The University of Auckland, Auckland, New Zealand; ^2^School of Humanities and Foreign Languages, Xi’an University of Posts and Telecommunications, Xi'an, China

**Keywords:** Butler, identity negotiation, Chinese gender discourse, Chinese academic women, photovoice interviews

## Abstract

**Introduction:**

This study employs Butler’s concept of *identity* to unpack how non-elite Chinese university academic women negotiate gender identities under the influence of the wider social discourses around gender in their institutional context.

**Methods:**

The study includes two empirical investigations of (i) semi-structured interviews and (ii) photovoice interviews with six women academics from six different non-elite Chinese universities.

**Results and Discussion:**

We found that most interviewees tended to value their teacher identity and meanwhile downplay their researcher identity in the professional field; while in the private field, they paid more attention to their mother identity at the cost of downplaying their career development. The study also reveals that, in the process of gender negotiation, the interviewees commonly adopted two strategies: (i) creating space and time for identity performance, and (ii) persuading with selves to accept multiple identities. This article contributes to the understanding of Chinese academic women in non-elite public universities from a Butlerian perspective.

## Introduction

Recent decades have witnessed an unprecedented rise in the numbers of employed women in all sectors of China. In the educational arena, official statistics show that the number of female academics slightly outstrips their male counterparts (Ministry of Education, 2020a). Gender equality seems to have been quantitatively achieved in Chinese higher education institutions (CHEIs). However, the increasing number of academic women has not translated into their elevation to higher-level academic positions. For instance, only 31.4% of academic women in CHEIs are in senior roles, such as associate professors, professors, deputy deans, deans, and presidents, and only 16% of doctoral supervisors are female (Ministry of Education, 2019). This indicates that women are still marginalized, as is the case worldwide. In literature, there is a dearth of research on academic women’s experience in non-elite CHEIs from the Butlerian lens (Zhao and Jones, 2018; Bao and Tian, 2022), despite the fact that non-elite universities are the mainstays of CHEIs quantitively.^1^ Most studies on academic women in China have focused on the general obstacles and challenges they face, but their multiple identities (teacher/researcher/mother), in both the professional and the private realms have rarely been explored.

This article thus fills this research gap by unpacking the subtle complexity of Chinese women academics’ negotiation of multiple identities. We focus on academic women in non-elite Chinese universities as their identities are often under-researched in literature and are, as found in our empirical data, strongly shaped by the institutional discourses and gender norms compared to that of women academics from elite Chinese universities (also see, Bao and Tian, 2022). Specifically, the study takes a Butlerian theoretical approach to interpret their experiences in non-elite universities because the Butlerian perspective is critical in deconstructing the existing gender relations in organizations shaped by wider discourses (Jenkins and Finneman, 2018). China is a country filled with both Confucianism discourses and contemporary discourse toward gender, which regulates individual behaviors and mindsets. Butlerian perspective thus offers valuable insights to investigate Chinese academic women’s experience in non-elite universities, specifically in this study, how these academic women’s identities are negotiated through conforming or subverting to gender norms, or in other words, doing or undoing gender. Although non-elite universities receive little attention in existing research, they represent the most common experiences of Chinese academic women. Therefore, enhancing understanding of this *invisible group* may support CHEIs to attract and retain the talents of academic women.

This study aims to examine Chinese academic women’s identities in both the professional and private realms of their lives and identify how mainstream discourses around gender exert an influence on their identity negotiation. Also, this might tell us about the external forces, the broader social discourses of the academy and gender in the Chinese context, heavily influencing the ways in which these academic women have negotiated with these identities they understand themselves to be in.

## Changing gender discourses in the Chinese context: A brief historical review

From feudal China of the pre-3rd century period dominated by Confucianism (Mak, 2013) to contemporary China, gender norms, shaped by external social discourses, have served as a crucial mechanism for proving the legitimacy of the prevailing regime and disciplining Chinese citizens (Barlow, 1994). The dominant discourse changes in different periods. This is not to say the new discourses replace the old ones; rather, the diversified discourses intersect, shaping gender norms and thus regulating individual behaviors and mindsets. We categorize the Chinese social discourses around gender into two main distinct periods, referring to the premodern period dominated by Confucianism, and the modern period dominated by marketization. We did so because there is a distinct shift in the social discourses around gender between the two.

In the premodern period, Confucianism played a vital role in shaping Chinese women’s status in Feudalism. Although Confucianism contributed to social harmony and stability, its gender views limited Chinese women’s status and agency in Feudalism and still heavily influence Chinese women today (Li, 2021). As Li (1997) states, “As long as it is still this nation that lives, breathes, and works on this stretch of land, it is impossible to be completely cut off from our history” (cited in Spakowski, 2011, p. 37).

Under Confucianist discourse, women are disciplined by “three obedience[s]” (*sancong*), that is, women must obey their fathers before marriage, obey their husbands when married, and obey their sons after their husbands die (see Gao, 2003). Meanwhile, women are excluded from education (Liu, 2014). The popular saying “a woman without talent is a virtue” (*nvzi wucai bianshide*), originated in the Qing Dynasty but is still being passed down by word of mouth. Besides, Confucianism also devalues women, as evidenced by the saying “only women and small-minded men are hard to feed” (*wei nvzi yu xiaoren nanyangye*). These gender views under Confucianist discourse limited Chinese women’s participation in education and public affairs and leadership in premodern Chinese society.

When China rolled into its modern period, marked by its socialist market economy reforms that began in the 1970s, its social discourses regarding gender also changed. On the one hand, the modern mass media heavily influenced femininity by publicly praising women for their femininity (Liu, 2014). On the other hand, the one-child policy improved urban girls’ status as their parents wanted to invest in their one and only child regardless of gender (Cai and Feng, 2021). Despite this, women were encouraged to succeed without losing their femininity (Liu, 2014). Gender differences and femininity thus became dominant ideologies (Sung, 2022). Popular discourses such as “men should focus on their careers, while women should focus on the family” (*nanzhuwai nvzhunei*), and “men should value their talent and women should value their appearance” (*langcai nvmao*) shaped Chinese men’s and women’s perceptions of the gendered division of labor. Such popular discourses, spread by mainstream media and even television series, have become the slogans that are now being passed around, shaping individuals’ mindsets and behaviors. Stereotypical gender discourses are widely accepted by the public and mass media in order to construct a harmonious society (Li, 2021).

In the 1990s, the wave of women being laid off due to the restructuring of state-owned enterprises became a social phenomenon, which accelerated women’s tendency to return home to be housewives and reinforced the reproduction of patriarchy. Yang (2020) argues that Chinese women are disadvantaged when their social and economic resources are restricted. Meanwhile, women with good appearances but lower levels of education were described as “flower vases” *(huaping)*. The objectification, in Butler’s words, *materialization*, of women’s bodies also regulates and restricts their desires and behaviors (Butler, 2011), making some of them more desirable for a successful marriage, while intellectual women with a high level of education were seen as the third sex^2^ that Chinese men try to avoid as their wives-to-be to maintain men’s superiority over women in household affairs and marriage (Liu, 2014; Dai et al., 2021).

By doing the historical review, we showed a wider Chinese context of how women’s gender mutates along with the changes in social discourses in the premodern and modern periods. In doing so, we introduced the wider discourses and social norms that enable us to examine and interpret the ways in which academic women in non-elite Chinese universities negotiate identities. In achieving the research purpose, Butler’s concept of *identity* was employed to unpack and interpret the subtle and complex experiences of Chinese academic women.

## Butler’s thoughts on gender identity

*There is no gender identity behind the expressions of gender; … identity is performatively constituted by the very “expressions” that are said to be its results* (Butler, 1990, p. 25).

Butler (1990) understands identity as a fluid and unstable process shaped by wider discourses and gender norms (also see Zhu and Chang, 2019). In most situations, gender norms function as a tool to discipline individuals and even as a source of influence on their self-recognition and identities (Butler, 2004). Butler also regards identity as “provisional,” an “error” or a “mistake” (1996, p. 324) as individuals may erase other identities in particular contexts.

Individuals can only be seen as intelligible by conforming to gender regulations and norms. As Butler states, “intelligible genders are those which in some sense institute and maintain relations of coherence and continuity among sex, gender, sexual practices, and desire” (1990, p. 17). Followed by this, intelligible academic women are those conforming to the gender and faculty norms and thereby maintaining the existing power relations in the academy, such as patriarchy and hierarchy. In this way, individuals tend to survive in the workplace by doing gender (Kelan, 2010).

Doing gender is a concept first theorized by West and Zimmerman (1987), which highlighted that gender is an important and continuous aspect of social interaction. Individuals purposively act on their gender identities based on what is seen as appropriate femininity or masculinity (West and Zimmerman, 1987). West and Zimmerman (2009) pointed out that doing gender works whether individuals conform or resist gender norms as individuals are at the risk of being judged to either side based on these norms. Hence, West and Zimmerman’s doing gender concept is difficult to interpret why individuals resist gender norms (Deutsch, 2007). More importantly, West and Zimmerman’s doing gender approach cannot help us to dismantle the existing gender relations. To address this issue, Butler’s (un) doing gender concept is slightly different, which suggested that one could also challenge the existing gender structures by undoing gender. Butler (2004) emphasized how the alternative performance could change the existing gender order. In other words, Butler’s theorizing could apply not only to interpret why individuals conform to gender norms but also resist them through subverting performance. Drawing on Butler’s view, Kelan (2010) indicates doing gender could be achieved through two approaches: women accept femininity, not performing in a masculine way; and women, especially those working in male-dominated discourses, conform to the visible or invisible gender norms and do not have a strong intention to challenge the existing gender structure. In this research, we borrowed the Butlerian perspective of *identity, discourse,* and *doing gender* to unpack academic women’s daily practices in CHEIs. The study contributes to gender research in CHEIs from a Butlerian lens and enhances understanding of the complex cultural and social *discursive nuances* in China that influence the complexity and plurality of the unstable *identities* of Chinese academic women and the ways in which they negotiated multiple identities through *doing gender*.

## Methodological considerations

In this study, purposive sampling was used as a method to recruit the six research participants, with the expectation that a small group of particular and invited participants can offer rich, detailed, and complex accounts of the topic (Braun and Clarke, 2006) and provide more in-depth information and understanding about the phenomenon under the investigation (Creswell, 2007). Specifically, we used our network of Chinese academics to invite volunteers who were interested in this research and then chose the most suitable ones to join the study. For the richness of the data, six academic women who were from the disciplines of Humanities and Social Sciences (HSS) at six different non-elite public universities were selected as the research participants. We recruited the participants from HSS as Chinese universities often undervalue HSS, that is, academic women in the HSS are at risk of being devalued and are therefore marginalized (Qi and Du, 2019). We chose non-elite public universities in China as the research site because they are the backbone of the Chinese higher education system. In this study, the academic role is defined as teaching and researching in non-elite public universities (*putong gongli gaodeng xuexiao*).^3^

Furthermore, this study only has a small scale of six participants because we did not aim to generalize academic women’s experience in non-elite universities; Rather, we are aiming to provide them with an opportunity for reflection on the negotiation of multiple identities in-depth. To fill this aim, we conducted two empirical investigations. The first investigation contained semi-structured interviews with six academic women (see Table 1) ranging from early stage to senior stage in non-elite universities in China as we would like to see the generational differences in their gender identities. Each interview was conducted face-to-face and individually, lasting approximately 1 h.

**Table 1:** The participants.

Pseudonyms	Age	Years of work experience	Academic rank	Degree	Marital status	Numbers of underaged children
Liang	38	4	Lecturer	Doctorate	Married	One
Huang	37	7	Lecturer	Doctorate	Married	Two
Liu	34	8	Lecturer	Doctorate^*^	Single	None
Zhao	44	22	Professor	Master’s^a^	Married	One
Fu	52	32	Associate professor	Master’s	Married	None
Chang	57	34	Associate professor	Master’s	Married	None

^*^denotes an international doctorate; other participants held a local doctorate.

a The late-career academic women in this study are Iron Rice bowl holders, which means they were permanently employed by the university (Si, 2022). In China, obtaining a doctorate is a bonus but not a prerequisite for their academic ranks, especially in non-elite universities. In the recent decade, Chinese universities transformed their human resource system from permanent to performance-based. This leads to a divide between late-career academics who obtained positions with a permanent contract and early-career academics who obtained positions under the publish-or-perish policy.

To research their identities within and outside academia, we asked them questions such as ‘How do you define your identity as an academic woman?’, ‘What pushes you to define your identity in this way?’, etc. Such questions help us to understand how wider social and cultural discourses shape academic women’s identities, thereby understanding the fluid process of identity negotiation. These interview questions are captured by the first research question: How do these academic women in non-elite Chinese universities negotiate their identities?

The second empirical investigation uses photovoice as the method to complement the first empirical investigation, aiming to answer the second research question: What strategies do these women utilize to negotiate with their multiple identities (teacher/researcher/mother)? Photovoice is a qualitative research method described by Wang and Burris (1994), who aimed to empower rural women by communicating with policymakers about rural women’s health needs in Yunnan Province, China. In the process of photovoice, participants are required to take photos of their lived experiences, followed by discussion and analysis with the researcher (Wang and Burris, 1997). The overall aim of photovoice is to give voice to marginalized and vulnerable groups, such as women, people with disabilities, and indigenous people (Wang and Burris, 1994). Informed by Wang and Burri’s work, some researchers and activists today recognized photovoice as a girl method that views women as knowers and actors (Moletsane, 2022).

Before the photovoice interviews, each participant was invited to take one photo which can show their negotiation with multiple identities. During the interviews, each participant was asked to share stories about the photo. The photovoice interviews were conducted three to 4 weeks after the first investigation because we wanted to give the participants sufficient time to delve into their lived experiences and prepare the photos, with each interview lasting approximately 45 min. Photovoice interviews enable us to gain further insights into the participants’ gender experiences (Wang and Burris, 1997). Also, the method gives the participants a chance to actively express the inner voice that they might find difficult to articulate and thus provide a richer and more complex experience that traditional methods cannot offer (Kara, 2015).

**Figure 1:**
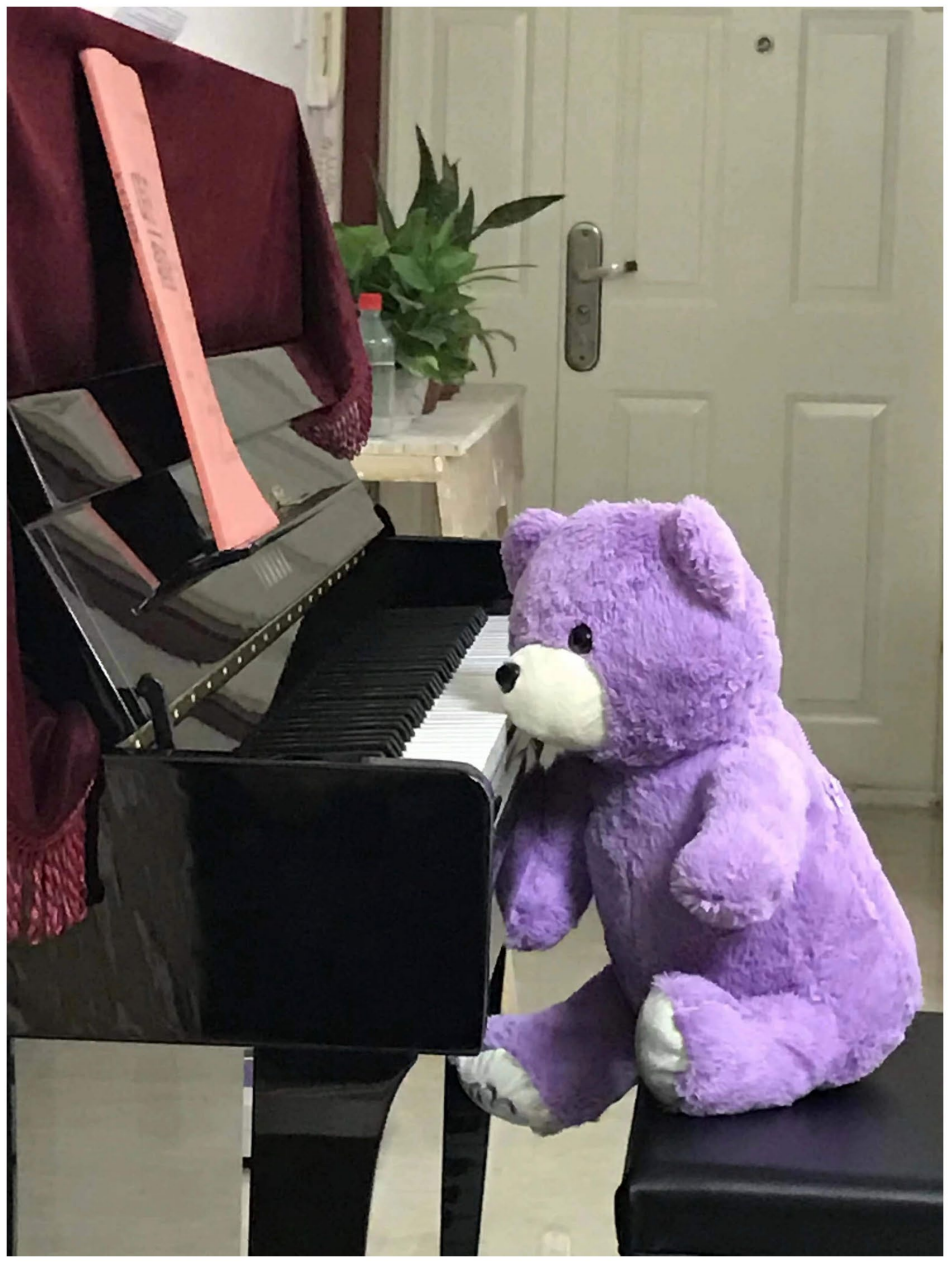
Liang’s photo (a long-time-no-use piano sitting beside a teddy bear).

After we analyzed the photovoice interview data, we found that the six photos share something in common. For example, Zhao’s photo of two pairs of shoes (a pair of high-heeled shoes and a pair of cotton slippers shows how she aimed to dress appropriately as a woman academic at work and relax to the utmost at home after work). Similar to Zhao’s, Liang’s photo of a piano (see Figure 1) can represent how she wanted to build up a harmonious relationship between her academic and mother identities, like playing the piano by coordinatingly using two hands. Given that the six photos shared some strategies in common, we chose to report only two of them in this paper that can facilitate our explanation of the two common strategies that the six interviewees used in negotiating with multiple fluid identities in their lives in a bid to avoid repetition.

Upon the completion of the two investigations, we conducted thematic analyzes of the qualitative data guided by Braun and Clarke (2006), with an emphasis on the ways in which the participants negotiate their multiple identities and the external social discourses underpinning such negotiations. We report on the findings in the next section.

## Identity negotiation in the professional field

The interview data suggest that, in the professional field, teacher identity was prominent and other academic identities such as researcher and leader were downplayed by the participants during the interviews. The self-proclaimed identity of being a good teacher appeared frequently in the accounts of the participants. Compared to becoming qualified researchers, many of them expressed their desire to be qualified teachers first. Even those who had been very good researchers gave weight to their teacher identity rather than their researcher identity. For example, Liang, who had been rated as an excellent researcher at the municipal level during her doctorate study, placed a higher value on her teacher identity rather than her researcher identity after working at the university, as recounted by her:

*After many years of working at the university, I think, compared to being an excellent researcher who is involved with inanimated research data most of the time, I prefer to be a good teacher who is engaged with live human beings every day. I enjoy the time talking with students and inspiring them when they are confused. I do this regularly, normally after lunch in my office. I am indeed into teaching in the classroom and inspiring students in the office.* (Liang)

Similarly, Zhao, who had already been a professor as of our interview, defined her identity in the professional field more as a good teacher instead of a successful researcher. When asked about something that made her feel self-achieved, she did not share her efforts and perseverance spent in the past 22 academic years becoming a professor as she was supposed to; rather, she shared one moment in relation to her teacher identity,

*Being rated as a professor does not impress me the most in my past academic experience. What makes me feel that I am a real educator with somewhat academically successful is my student. Upon graduation, a student wrote me a long letter, saying that one thing in which the university had benefited him the most was related to me. According to the letter, I had helped him develop very good reading and thinking skills and habits, which further enabled him to be a successful graduate of the university with first-class honors. So far, I am still very touched and moved by his words*. (Zhao)

In Liang’s case, she identified herself as a teacher for students, although more time spent on her teacher identity may have limited her time and energy invested in her researcher identity under the contemporary climate of CHEIs. Since 2013, CHEIs have started a “publish or perish” system to evaluate academics’ performance with the key indicators of publication and project grants (Li and Shen, 2020). Liang’s university also adopted this system. Liang signed a “publish or perish” contract with the university in which she worked, which means she may lose her job if she cannot survive the research grant application or achieve the required number of publications in a certain time frame. In addition, the heavy workload in teaching carried by Liang may also limit her time spent on research, thereby influencing her upward academic career mobility (see, Thomas and Davies, 2002). Under such competitive conditions, Liang still prioritized her teacher identity. In the Butlerian view, Liang’s recognition of her teacher identity was enacted through “the forced reiterations of norms” (McKinlay, 2010, p. 235) – these social norms are deeply rooted in China’s traditional cultural heritage of Confucianism and modern secular social discourses.

China has a long Confucian tradition of “respecting teachers and valuing education” *(zunshi zhongjiao)*. This discourse has exerted a strong influence on academic women’s negotiation of their professional identities, including Liang and Zhao. Moreover, we find that the participants frequently shared their desires to be good teachers and educators respected by students, rather than to be excellent researchers. For them, “*being a good teacher respected by students can bring in a sense of achievement*” (Zhao’s words) even though they were working in universities where the key performance indicators of teacher evaluation devalued teaching workload and prioritized research outputs (see, Morley and Crossouard, 2015).

The two cases of Liang and Zhao demonstrate the teachers’ negotiations with the institutional discourses of the universities (e.g., performance-based evaluation system)^4^ and the traditional social discourses of Confucianism (e.g., a woman without talent is a virtue; men should focus on their careers, while women should focus on families) However, the contradictory discourses leave Liang and Zhao in a dilemma. After the process of identity negotiation, they determined to keep their teacher identity in the professional field and downplay their researcher identity. In this sense, gender discourses shaped by Confucianism have a heavier impact on non-elite academic women’s identity negotiation than the institutional discourses of the universities. Similarly, Eagly and Sczesny (2019) argued that gender exerts a heavy influence on daily life even when competing with the influences of other social roles. Their teacher identity, enhanced by daily practices, maintained the gender norms and gender division of labor in contemporary Chinese society. This contradicts Clegg’s (2008) research in the UK, where academic women in non-elite universities still passionately talked about their researcher identities, although they did not prioritize their research role.

The two cases demonstrate that non-elite Chinese university academic women’s identity is negotiated through doing gender (Liang and Zhao). In the academic sphere, teaching is regarded as a more feminine thing whereas males are often expected to invest much more time and energy in research, rather than bearing in mind many teaching and family demands (Fairhurst, 2009). This is also an invisible institutional norm in CHEIs, which treats the research work as masculine and teaching as feminine. Under such conditions, academic women participants like Liang and Zhao comply with the gender binary and social norms by doing gender in order to be acceptable and intelligible. As Jenkins and Finneman (2018) state, those who do not comply with gender norms are “ostracized within the culture, as the broader power structure prioritizes the maintenance of a gender polarity or binary” (p. 159).

However, there was an exception in the interviews. Liu, who had a great ambition for academic career mobility, defined her identity in the professional field more as a researcher. She started her doctorate study overseas and meanwhile retained her work in a non-elite Chinese university. She immersed herself in an environment filled with academic research. Unlike other participants, when asked about her favorite aspect of being an academic woman, she emphasized the role of doing research, as she said,

*Doing research and teaching is tightly wound together. Intensive research work can offer lots of inspiration for teaching. Also, the reward of research is more obvious. The more time and energy you spend on the research, the more reward you can get. The return of research is visible, but that of teaching is not.* (Liu)

Liu was more confident than other participants in doing research partly due to her singlehood. She was single as of the interviews and was not in want of getting married; as she said, ‘*being single is a choice I made by my own initiative as I do not want marriage to limit my career and personal growth*.’ Liu treated marriage as a barrier to her success and, in the meantime, associated singleness with self-development and achievement. However, Liu’s choice of singleness makes her distant from other academic women who see having a family as normal for women. As she mentioned, ‘*Many of my female colleagues share their family and parenting experiences, I always seem out of place at this time because I have nothing to share about this topic. I have a personal feeling that I look strange in the eyes of these female colleagues’*. In the Chinese context, individuals are expected to ‘do correct things at the correct time’, which regulates their personal life and choice. For Chinese women, in particular, marriage and childbirth at the right age are in line with these social expectations. Therefore, Liu is recognized as not intelligible by the wider society due to her personal choice of being unmarried and concentrating on her career.

Her identification of herself as a researcher is also partly because of her professional doctoral training overseas. As she said, “*studying abroad offers me confidence and rich experiences in conducting research.*” Therefore, she preferred to identify herself more as a researcher. Furthermore, she believed that “*the metric evaluation of a researcher is visible because the research grants and the number of publications is accountable, while teaching is labor-intensive work with uncertain rewards.*” (Liu’s words). In this way, her researcher identity was negotiated by the interplay between her singlehood, her overseas doctoral experiences, and her understanding of the key performance indicators required by the university where she worked.

Liu’s narrative shows her identity negotiation is achieved through undoing gender. She adopted the metric evaluation which is exercised through masculine norms, which define an ideal academic person as “someone with unlimited resources available to dedicate to work” (Fox, 2020, p. 214) and “unwavering commitment to the organization” (Bierema, 2016, p. 119). However, the discourse about an ideal academic person does not take women’s experiences and reality into account, seen in that academic women are strongly expected to spend more time teaching students as caring teachers and meanwhile taking care of their families and children as virtuous household wives. Under such conditions and to survive the professional field, Liu described how she valued her researcher identity by undoing gender (Butler, 2004), that is, abandoning her femininity to conform to masculine norms. However, Liu’s negotiation is not recognized by the wider gender norms. She would rather not be intelligible to society than defends her identity as a researcher in the professional field.

## Identity negotiation in the private field

Our investigation into the private field of the research participants reveals that social discourses around gender have influenced their negotiation of identity as academic mothers. For example, one participant, Chang, who started her career as a university lecturer during the period of China’s socialist market economy reforms in the 1980s, had considered changing to a job outside academia at the outset of her academic career. However, this consideration ceased after the birth of her daughter. As she narrated,

*During the beginning two years of working at the university, I wanted to change my job and achieve something outside academia. However, later, my mind changed after I got married and delivered my daughter, which made me very busy meeting the household and family demands, and which also changed me and I realized that family is more important than an academic career. For now, I don’t even think about investing time and energy in academic career development.* (Chang)

Chang expressed a desire to achieve something outside academia before getting married and becoming a mother because the market economy reforms in China in the 1980s had created lots of promising job opportunities outside academia (He and Wu, 2017). These reforms also made many men unwilling to find academic jobs that were less well-paid than jobs in other social sectors and undertakings where higher salaries were paid for high-skilled workers (Tang and Horta, 2021). As for Chang, although a more promising job opportunity was abandoned, she believed that she had made “*the best choice to be an academic*” in the 1980s because, in her words, “*choosing to be an academic gives [her] time and energy to take care of [her] family.”* That is, by abandoning the possibility to work outside academia for keeping her caring mom identity in the social discourse.

Chang’s choice and explanation also indicate that she has yielded to the existing gender discourses and has accepted her identity as a caring mother in the private field. According to Confucian tradition, a ‘virtuous wife and caring mom’ (*xianqi liangmu*) are more expected by society. In addition, in China’s context, compared to working outside the university, staying in academia gave her more room to take care of her family and daughter as being an academic woman has more flexible time and energy to attend to the burden of family demands. While Chang initially desired to leave academia to pursue another career path, the normative behavior shaped by gender discourses (such as being a caring mother) called her back to the grid of intelligibility. Consequently, by giving up the desire to start another career, Chang negotiated her identity as an academic mother, which was, in turn, a way to maintain social and gender norms through doing gender (Butler, 2004).

Chang’s case shows that women, to an extent, must compromise with the gender norms that have already existed in a bid to be intelligible and socially accepted. This phenomenon is becoming increasingly pervasive for academic women in China and is not only limited to Chang, as mentioned by another participant, Fu:

*When I was young, I believed women can achieve whatever men can achieve. However, as I became older, I have to admit that, compared to women who are expected to put more time and energy into household issues while working, men are privileged and advantaged in career development. As for me, I have paid too much time and energy to become an associate professor, thus I have been absent too much from my daughter’s childhood. This is one of my permanent regrets.* (Fu)

In this case, Fu made a judgment about men and women based on the patriarchal structures of Chinese society, implying women are disadvantaged in the professional field compared to men because they are presumed to take more responsibilities for family and children in the private field. This has already been rationalized by many senior stage women academics such as Fu, who refused the existing gender power relations at an early age. Additionally, Fu assumed her academic identity (being an associate professor) was at the cost of erasing her motherhood whereas she blamed herself for downplaying mother duties (the companionship in her daughter’s childhood) to gain her academic career success and regarded this as her *permanent regret*.

The traditional Chinese social discourse of caring mom has had a strong influence on Fu’s behaviors and mindset, although she did not totally conform to it in her early stage period. Moreover, she did not realize that men in most cases do not suffer such expectations to be present for their children. Thus, rather than criticizing men who often spent more time and energy in the workplace instead in the family, Fu accepted and internalized the social discourse that females should take on more household responsibilities for taking good care of children. As Butler (1990) explains, individuals are not free to choose their identities but perform a preexisting structural identity shaped by contextualized discourses. These regulatory regimes and discourses within and outside the academy, in Butler’s (1990) words, *hail* (summon) women academics back to the intelligible matrix, in which their identities seem to remain stable and fixed.

## Identity negotiation: Common strategies used by the participants

Academic women’s negotiation with different identities (teachers/researchers/mothers) was a theme raised by the participants in the second empirical investigation which used photovoice as the data collection method. The data suggest that women’s professional identities (teachers/researchers) sometimes overlap with their private identities (mothers); this adds double burdens for the participants. We do not extend our explanation here given that this finding has been pervasively discussed in the existing literature (e.g., Manathunga et al., 2020; Xian et al., 2021). Rather, our focus is given to the strategies that the participants used to negotiate with multiple identities, which might shed light on women who are fighting for their identity rights (Sung, 2023).

As explained in the foregoing methodological section, two participants’ photos were, respectively, utilized here to demonstrate the two common strategies of identity negotiation found through the data. They are (i) creating time and space for identity performance (as exemplified by Huang’s case below), and (ii) negotiating with the self to accept multiple identities (as exemplified by Liang’s case below).

*I want to share the two photos together because they can represent my arrangement of both space and time after class. I don’t like working in the office because there are more than ten colleagues crammed into one office with limited space for each person. Only academics with the title of professor and leadership were assigned a personal office. This gives us opportunities to work in a third place without any interruption. The café shows my working space, and the watch shows my fragmented time. I put them together because academic women like me often need to deal with space and time management after class. I have to frequently answer household calls after class. This café is close to my place, which is an ideal place for me to work after class. The family atmosphere with endless chores and noisy children leaves me no room to work after class. Therefore, I work and write in this café, as many of my female colleagues do. We all feel that the café provides an ideal space for us to stay. The cozy environment with decent coffee and dessert here makes me highly energetic. It’s hard to see male colleagues work here. They often work from the office or home mainly because they don’t have to spend money in a café like this. For me, time management counts most because I undertake most of the chores and childcare. The time that I can manage in this café is very limited, so I no longer use my cellphone here so as to avoid being interrupted by other daily business. If I have to check the time, I prefer to use this watch*. (Huang)

**Figure 2:**
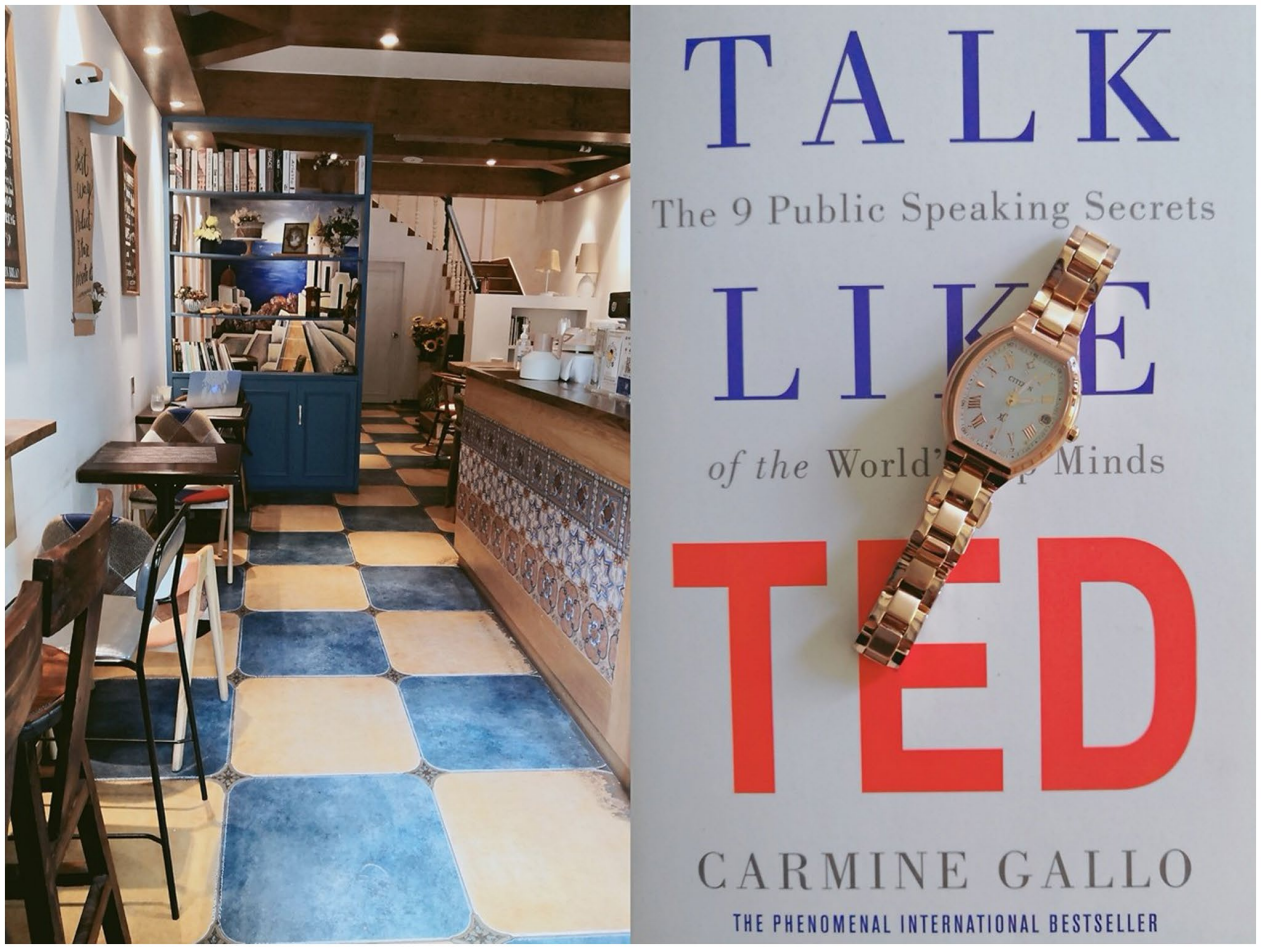
Huang’s photos (a local café and her watch).

Figure 2 shows Huang’s strategy to negotiate with her multiple identities. She put the two photos together because she could not separate time and space, which are valuable for academic women. Huang, as a mother of two children, carried the burden of the majority of the household demands. Although she had been accepted by an elite university in China, she eventually chose to work at a non-elite university that was closer to her home –this gives her more time and room to take good care of her family.

Huang’s choice was influenced by the wider Chinese social discourses and environment. The Chinese public often criticizes women who are ambitious in the workplace at the cost of downplaying their families (Liu, 2014) and promotes the traditional standardized image of the ideal mother’s identity as a caring mom who devotes both time and energy to their children with regretless. In this way, the Confucian discourse on gender identity hailed academic women into place in private realms of life, encouraging them to continue to keep their femininity and motherhood. This can also explain why some well-educated women who graduated from elite universities chose to work in non-elite ones.

Huang’s other identities, such as being a productive researcher, were thus hidden in the private field. This is understandable because “when we act in certain ways as women in particular contexts, other expressions are always silent, erased, hidden — to ourselves and others” (Jackson, 2004, p. 677). Especially since the two-child policy was gradually introduced and enacted in China, motherhood has added an extra burden for academic women because they have more children to take care of (Shen and Jiang, 2021). Under such conditions, the café was an ideal place for Huang to work after class. Only in the café did Huang feel that her identity as an academic woman was fully played because she could devote herself to her academic work, without negotiating with her other identities.

Huang attributed the lack of male colleagues working in the café to their unwillingness to spend money. However, other reasons for males working from either office or home are hidden. For instance, men can work at the office or from home in a carefree manner if their wives can undertake the entirety of domestic chores and childcare responsibilities. This also implicitly shows Huang’s internalization of gender discourses in the Chinese context. In Huang’s case, changing working space, quitting the mobile phone, and using a watch to check the time were her tactics for navigating different identities, such as mother, wife, and academic. Huang’s photos show her strategy to negotiate with both her mother identity and academic identity.

*I used to learn to play the piano when I was a secondary school student. I enjoyed playing the piano and dreamed of having my own piano. After marrying, I eventually owned a piano. But I have yet had no time to play it because I have either been busy with work or with taking care of my family. I put a bear there. It seems that another me was playing the piano. This piano carries my wish, but I can’t fulfill it in reality. Additionally, playing the piano well requires hand and foot coordination, just like my work and life, and I’m not willing to abandon any of it, so I must become a “good piano player” in my everyday life.* (Liang)

By using the photo and the piano metaphor (as shown in Figure 1), Liang gave an account of the strategy in which she negotiates with her multiple identities (caring teacher, researcher, busy mother) that contradicted each other. The desire to become a good piano player shows her resolution to perform each of her multiple identities well. Her negotiation with multiple identities at that moment was influenced by discourses around gender norms in both the professional and private fields. In this case, Liang tended to identify herself as a gender-neutral academic at work, not a woman. Meanwhile, she also desired to keep her feminine gender identity at home. It seems Liang made great efforts to perform each identity well. However, she failed to do anything to subvert her different identities at present but to conform to gender norms in each field, in order to accommodate a role that is more enriching. In this sense, Liang’s process of identity negotiation was achieved through doing gender.

Both cases (Huang and Liang) demonstrate their individualist strategies to negotiate with multiple identities by doing gender (Butler, 2004). Specifically, Huang tended to create a space and time in which to perform her academic identity while Liang chose to adjust her mindset to settle her multiple identities, influenced by institutional and gender norms. Both cases demonstrate academic women’s individualist strategies for handling some tensions in both the professional and private fields. It seemed that they were very resilient; however, they might have fallen into the identity claims trap in order to be intelligible, that is, they always changed their behavior to fit into external social discourses rather than attributing their identity struggles to the problematic effects of the existing social structures (Shepherd, 2017).

## Conclusion

This study offers a Butlerian lens to investigate academic identity negotiations in both professional and private fields. We argue that Chinese gender discourses have a significant bearing on the six non-elite Chinese university academic women’s negotiation of different identities. They valued their teacher identity in the professional field, but most of them kept a distance from their researcher identity. This resonates with Bosanquet’s (2017) finding that academic women are overloaded with teaching duties. Instead of pursuing higher academic ranks as their priority, the academic women in this study spent more time teaching and supporting students. Furthermore, the participants who have already been mothers valued their motherhood by showing their willingness to downplay their academic careers to preserve their intelligible gender identity as household responsibility bearers in the private field. For example, Fu treated her neglect of motherhood duties as shame and failure in her daughter’s childhood after self-reflection. This also can explain why Liang desired to be a good piano player who was capable of juggling responsibilities and commitments between different spaces and times and performing multiple identities successfully.

The findings in this study also resonate with existing literature on academic women’s negotiation of multiple identities in other parts of the world. For example, Bosanquet (2017) found that Australian academic women chose to reduce sleep time or even consider leaving academia after gender negotiation; the strategies academic women utilized are filled with sacrifice, similar to that of the academic women in this study. The normative expectations of women resulted in academic women’s negotiation of how they should perform, and most of them chose to be silenced in the faculty (Aiston and Fo, 2020). The negotiation between femininity and professional identity enables women to be caught in a paradox – the characteristics that help them succeed at work tend to contradict the roles they have to fulfill in the private field (Cherkowski and Bosetti, 2014).

The academic women in this study utilized some individualist strategies to cope with multiple identities for performing each identity well. In this study, we found that most of the interviewees tended to utilize different strategies to assist their doing gender in non-elite universities, such as making a café a third space between the workplace and the family place for coping with academic stuff after school. Their purpose was to fit into the academy and yet also performed gendered roles recognized by society. In women’s efforts to negotiate with different identities, they defied the traditional social discourse around gender – “a woman without talent is virtuous” (*nvzi wucai bianshide*) (Liu, 2014). However, the individualist strategies adopted by these women also demonstrate their adaption to the existing social and institutional structure and discourses, rather than challenging them, which may further aggravate the gender dilemma of academic women in both professional and private situations.

This study also reveals some generational commonalities and differences in identity negotiation between early stage and senior stage academic women. We found that both groups of academic women, negotiated their multiple identities through doing gender, that is, conforming to the social discourse and gender norms, aside from Liu (an academic beginner). Liu voluntarily abandoned marriage to achieve personal and career success from her definition of what it means to be an academic woman. This also suggests that not every academic woman chose to negotiate with their multiple identities through doing gender. The major difference between the two groups can be seen in that early stage academic women desired to perform each identity well whereas senior stage ones tended to prioritize their mother identity in the private field. It should also be worth noting that senior stage academic women in this study such as Chang and Fu, only retrospectively reflect their duties to families. Tensions between professional and private fields may be less intense when their children become adults.

Hence, we conclude that the academic women in this study tended to internalize the gendered discourse implicitly or explicitly. They more or less expressed pressure through doing gender; however, they took such pressure and struggle for granted, which was harmful to their well-being and happiness (Butler, 2004). In line with Manathunga et al.’s advocation, we should seek to rebirth “a new kind of academy that will support – rather than penalize – academic women (and men) in their commitments and responsibilities for the care of others” (2020, p. 250).

This study contributes to unpacking a small sample of Chinese non-elite academic women’s identities negotiation through the Butlerian perspective of identity and gender. However, there are also limitations in this study that should be clarified. Firstly, Butler suggests that there are possibilities to subvert gender identities individually and collectively through undoing gender (Jenkins and Finneman, 2018). However, the participants in this study did not deviate from gender norms in their negotiation of multiple identities, which does not represent all Chinese academic women’s experiences at both elite and non-elite universities. Future studies may investigate how academic women subvert their identities by undoing gender. Secondly, the small sample of non-elite academic women in this study cannot represent all Chinese academic women’s negotiation of identities and strategies of handling due to the exploratory nature of this study but do offer new insights to understand how academic women conform to gender norms after the process of identity negotiation. Future studies may add more diverse academic women such as academic women from elite universities to understand academic women’s identities.

## Data availability statement

The raw data supporting the conclusions of this article will be made available by the authors, without undue reservation.

## Ethics statement

The studies involving human participants were reviewed and approved by the ethics committee of the University of Auckland. The patients/participants provided their written informed consent to participate in this study.

## Author contributions

BY: conception and design of the study, conducted the data analysis, and wrote the first draft of the manuscript. XT: validated the data analysis and revised the manuscript. All authors contributed to the article and approved the submitted version.

## Conflict of interest

The authors declare that the research was conducted in the absence of any commercial or financial relationships that could be construed as a potential conflict of interest.

## Publisher’s note

All claims expressed in this article are solely those of the authors and do not necessarily represent those of their affiliated organizations, or those of the publisher, the editors and the reviewers. Any product that may be evaluated in this article, or claim that may be made by its manufacturer, is not guaranteed or endorsed by the publisher.

## Footnotes

^1^According to official statistics released by the country’s Ministry of Education, there are 1,265 universities in China offering undergraduate degrees. Among them, 137 are elite public universities and 257 are private universities (Ministry of Education, 2017, 2020b). This means that at least 871 universities are non-elite public universities, which owns the highest number of students in China.

^2^Third sex is the stigmatization of women PhD as asexual and nonfeminine, see Kuo (2014).

^3^Non-elite universities here refer to public universities that are not on China’s double first-class university list. In 2017, China’s Ministry of Education launched an educational plan, selecting 137 universities from the country’s thousands of higher educational institutions to construct them into either first-class universities or universities with first-class disciplines at the world level.

^4^In order to speed up the building of world-class universities, Chinese universities gradually reformed its policies related to faculty recruitment and promotion from contract-based to performance based. This means research activities are prioritized over teaching and other duties (Si, 2022). This is more evident in elite universities. Numerous Chinese non-elite universities, however, have a tendency to adopt this system but have not yet fully realized it due to the large number of demands for staffs (Lai, 2012).
